# The effects of water temperature on gastric motility and energy intake in healthy young men

**DOI:** 10.1007/s00394-018-1888-6

**Published:** 2019-01-07

**Authors:** Kyoko Fujihira, Yuka Hamada, Takuma Yanaoka, Ryo Yamamoto, Katsuhiko Suzuki, Masashi Miyashita

**Affiliations:** 1 Graduate School of Sport Sciences, Waseda University, 2-579-15 Mikajima, Tokorozawa, Saitama 359-1192 Japan; 2 Japan Society for the Promotion of Science, 5-3-1 Koujimachi, Chiyoda-ku, Tokyo 102-0083 Japan; 3 Faculty of Sport Sciences, Waseda University, 2-579-15 Mikajima, Tokorozawa, Saitama 359-1192 Japan

**Keywords:** Water ingestion, Water temperature, Gastric motility, Ultrasound imaging, Appetite, Energy intake

## Abstract

**Purpose:**

Although immediate pre-meal water ingestion has been shown to reduce energy intake in healthy young men, no studies are available regarding potential mechanisms underlying the effect of energy intake in response to different temperatures of pre-meal water ingestion. This study examined the effects of consuming different temperatures of water on gastric motility and energy intake in healthy young men.

**Methods:**

Eleven young men were completed three, 1-day trials in a random order. Subjects visited the laboratory after a 10-h overnight fast and consumed 500 mL of water at 2 °C, 37 °C, or 60 °C in 5 min. Then, subjects sat on a chair over 1 h to measure the cross-sectional gastric antral area and gastric contractions using the ultrasound imaging systems. Thereafter, subjects consumed a test meal until they felt completely full. Energy intake was calculated from the amount of food consumed.

**Results:**

Energy intake in the 2 °C (6.7 ± 1.8 MJ) trial was 19% and 26% lower than the 37 °C (7.9 ± 2.3 MJ, *p* = 0.039) and 60 °C (8.5 ± 3.2 MJ, *p* = 0.025) trials, respectively. The frequency of the gastric contractions after 1-h consuming water was lowered in the 2 °C trial than the 60 °C trial (trial-time interaction, *p* = 0.020). The frequency of gastric contractions was positively related to energy intake (*r* = 0.365, *p* = 0.037).

**Conclusions:**

These findings demonstrate that consuming water at 2 °C reduces energy intake and this reduction may be related to the modulation of the gastric motility.

## Introduction

Public health research is addressing on the long-term management of weight loss by creating a negative energy balance through increased physical activity and/or decreased food intake in overweight and obese individuals [1]. However, estimates in many countries suggest that most individuals do not complete a sufficient amount of physical activity to meet the guidelines set out by expert panels [2, 3]. Thus, while promoting physical activity for all individuals is important, strategies to prevent a positive energy balance and subsequent weight gain in healthy individuals may be important for the long-term weight management. Among available methods for preventing a positive energy balance, water consumption is a simple method and has potentially a key role to play in reducing energy intake [4].

To date, three laboratory-based studies have examined the effects of pre-meal water ingestion on subsequent energy intake in various individuals [5–7] with disparate effects. These studies vary in protocols including the amount of water ingested (i.e., 375–568 mL), and the time interval between ingestion of water and the subsequent meal (i.e., immediately before to 30 min before a meal). In addition, only one study has clearly reported the temperature of water used in the study (5–7 °C) [7]. Although the reasons for these discrepant findings among studies are not clearly known, Corney et al. [6] have suggested that the rate of gastric emptying of liquid meals was slower in older adults than in younger adults, indicating that gastric distension may be a factor for influencing subsequent energy intake. In addition, the rate of gastric emptying or the magnitude of gastric motility (i.e., measured via cross-sectional antral area reflecting gastric distention, rate of gastric emptying, and frequency of gastric contractions) are known to be influenced by the temperatures of consumed “energy-containing drinks” [8, 9]. Collectively, to our knowledge, none of previous studies [5–7] have examined the effects of pre-meal water ingestion on gastric motility and subsequent energy intake in healthy young adults. Furthermore, there is as yet no evidence regarding how different temperatures of “water” affect energy intake and subjective feelings of appetite in healthy young adults.

Therefore, the purpose of this study was to investigate the effects of different temperatures of water on gastric motility and energy intake in healthy young men.

## Methods

### Subjects

After approval from the Ethics Committee on Human Research of Waseda University (approval number: 2017-260), 11 healthy, lean men gave written informed consent to participate in this study. The physical characteristics of the subjects were as follows: age 23.4 ± 1.4 years, height 1.71 ± 0.04 m, body mass 64.0 ± 9.8 kg, body mass index 21.8 ± 2.6 kg/m^2^, and waist circumference 73.1 ± 5.4 cm [mean ± standard deviations (SD)]. All subjects were non-smokers and were not taking any medicine, and their body masses had been stable for at least 3 months before the study.

### Experimental protocol

The subjects underwent three, 1-day laboratory-based trials in random order: (1) water at 2 °C, (2) water at 37 °C, and (3) water at 60 °C. The interval between trials was at least 6 days. All subjects were asked to maintain their normal eating habits among the trials and to refrain from vigorous exercise and alcohol intake for 24 h before each trial. In the 24 h before the first trial, subjects measured and recorded all dietary intakes, and then they replicated these dietary intakes in the 24 h preceding the second and third trials. Food diaries were analyzed by software to determine energy intake and macronutrient content (Excel Eiyoukun Ver 5.0, Kenpakusha, Japan). On each trial day, subjects reported to the laboratory at 0850 after a 10-h overnight fast-subjects were allowed to drink only one glass of water no later than 2 h prior to each trial. The subjects were asked to sit on a comfortable chair in a fixed position (i.e., the angle between the upper part and lower part of the body was approximately 120°) and to consume a 500 mL of water at 2 °C, 37 °C or 60 °C over a 5-min period at 0900. The water temperature was measured by an electric thermometer (testo 106, Testo K.K., Japan). The 500 mL of water was chosen, because this volume has been shown to reduce subsequent energy intake in a previous study [7]. Subjects then sat on a chair in a fixed position as above in the laboratory until 1005. Whilst subjects rested until 1005, 2D ultrasound scan was performed to assess the change in the cross-sectional gastric antral area and gastric contractions before and after consuming a 500 mL of water at 2 °C, 37 °C, or 60 °C. Then, subjects were asked to consume the test meal from 1005 and were instructed to eat as much as they satisfied until 1105. The interval of 60 min between water ingestion and subsequent meal was chosen, since we thought that this interval may be long enough to assess gastric motility using ultrasound imaging as this was the case in the previous study [10]. Subjects also completed a 100-mm visual analogue scale questionnaire [11], assessing the subjective perceptions of appetite, at 0900 (i.e., pre), immediately after consuming water (i.e., post), and 30, 60, 90, and 120 min after consuming water.

### Subjective appetite perceptions and energy intake

The acceptability of the test meal was ensured by a prior written survey and selected instant noodles as a test meal. Subjects were provided with a bowl of instant noodles (i.e., 9.7% energy as protein, 20.5% energy as fat, and 69.8% energy as carbohydrate) at 1005. Subjects were offered with repeated small bowls of noodles throughout the meal time. Warm food was continuously available until the subjects finished eating the test meal. Subjects were instructed to eat until they felt “comfortably full and satisfied” and that additional food was available if desired [12]. This ensures that subjects were not able to know how much they had consumed while eating. Drinking water was restricted, while the subject was taking the test meal. The upper limit of meal intake time was 1 h, and mean time to consume the test meal in the 2 °C, 37 °C, and 60 °C trials was 18.2 ± 6.5 min, 21.8 ± 9.4 min, and 24.1 ± 11.9 min, respectively. The total amount of food intake (g) was ascertained by examining the weighted difference in the test meal remaining compared to that initially presented. The total energy intake from the test meal was calculated using the manufacture-reported values. Subjects completed 100-mm visual analogue scales [11, 13] before (i.e., pre), immediately after (i.e., post), and at 30, 60, 90, and 120 min after consuming water, to assess the perceptions of appetite (i.e., hunger, fullness, and desire to eat sweet, sour, fatty, and salty foods). In addition, subjects completed 100-mm visual analogue scales before (i.e., pre), immediately after (i.e., post), and at 30, 60, 90, and 120 min after consuming water, to assess the perceptions of feelings of stomach condition (i.e., “Does your stomach feel uncomfortable?”, “Do you feel your stomach is expanding?”, and “Do you want to eat now?”). Verbal anchors “not at all” and “extremely” were placed at 0 and 100 mm on the visual analogue scales, respectively.

### Assessment of gastric motility

Several previous studies suggest that the antrum is the most suitable area in which to evaluate the stomach capacity (for a review of this, see Ref. [14]). Antral area measurements were performed using a 2D ultrasound machine (LOGIQ-e, GE Healthcare, USA) and a 5.0 MHz sector transducer. All metals were removed from the surrounding area to avoid the possibility of interference during acquisition. To optimise precision, the transducer was positioned vertically to obtain a parasagittal image of the antrum, with the superior mesenteric vein and the abdominal aorta in a longitudinal section, as described previously [14]. After obtaining these signals for measuring antral area for 3 min [15] before (i.e., pre), immediately after (i.e., post), and at 10, 20, 30, 40, 50, and 60 min after consuming water. The gastric antral area (cm^2^) was determined using an image-editing software (ImageJ 1.47, National Institute of Mental Health, USA). The gastric contractions of the antral area were defined as the frequency of contractions per 3 min, and were measured before (i.e., pre), immediately after (i.e., post), and at 10, 20, 30, 40, 50, and 60 min after consuming water [16].

### Statistical analysis

Data were analyzed using the Predictive Analytics Software (PASW) version 23.0 for Windows (IBM SPSS Statistics 23.0, SPSS Japan Inc., Japan). The Shapiro–Wilk test was used to check for normality of distribution—all parameters were found to be normally distributed. Repeated-measures one-factor analysis of variance (ANOVA) was used to assess differences among the three trials in energy intake and the length of meal. Repeated-measures, two-factor ANOVA was used to examine differences over time among the three trials in cross-sectional antral area, frequency of gastric contractions, subjective appetite perceptions (i.e., hunger, fullness and desire to eat sweet, sour, fatty, and salty foods), and subjective perception of the stomach. Where significant trial–time interactions and trial effects were found, the values were subsequently analyzed using a Bonferroni multiple-comparison test. The correlation coefficients were determined using Pearson’s product-moment tests between the frequency of gastric contractions and energy intake. The 95% confidence intervals (95% CI) for the mean absolute pairwise differences among the three trials were calculated using the *t*-distribution and degrees of freedom (*n* − 1). Data were expressed as mean ± standard deviations (SD). Statistical significance was set at *p* < 0.05.

## Results

### Dietary data

Mean self-reported energy intake for the day prior to each trial was 7.2 ± 1.5 MJ. Energy intake equated to 30 ± 6% (59.9 ± 20.7 g/day) from fat, 54 ± 7% (218.3 ± 30.8 g/day) from carbohydrate, and 16 ± 3% (70.4 ± 20.6 g/day) from protein.

### Pre-trial

There were no significant differences in body mass among the 2 °C, 37 °C, and 60 °C trials (63.8 ± 9.9 kg vs. 64.3 ± 9.7 kg vs. 63.9 ± 9.9 kg, respectively; *p* = 0.075) at pre-trial (i.e., 0900). At pre-trial, subjective appetite perceptions (i.e., hunger, fullness, and desire to eat sweet, sour, fatty, and salty foods) and perception of the stomach did not differ across the trials. Cross-sectional antral areas (3.1 ± 1.3 cm^2^ vs. 3.6 ± 1.3 cm^2^ vs. 3.1 ± 1.0 cm^2^ for the 2 °C, 37 °C, and 60 °C trials, respectively; *p* = 0.277) and frequency of gastric contractions (4.5 ± 2.5 times/3 min vs. 4.5 ± 2.7 times/3 min vs. 4.5 ± 3.0 times/3 min for the 2 °C, 37 °C and 60 °C trials, respectively; *p* = 0.996) were also not different at pre-trial (i.e., 0900) among the trials.

### Energy intake

**Fig. 1:**
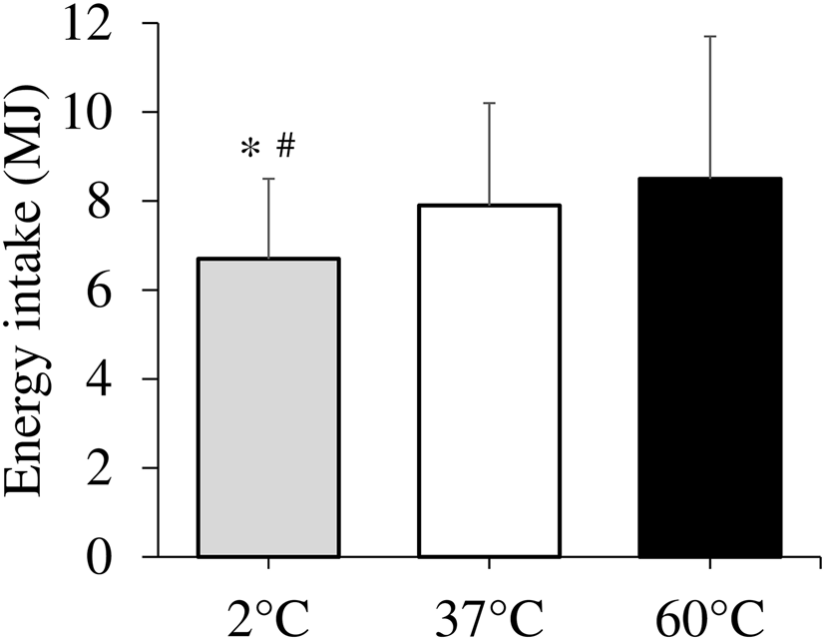
Energy intake at ad libitum test meal: 60 min after consuming water (500 mL) at 2 °C, 37 °C and 60 °C. Data are mean ± SD. Mean was compared using one-factor ANOVA for the main effect of trial followed by a Bonferroni multiple-comparison test. *Significantly different between the 2 °C and 60 °C trials (*p* < 0.05). ^#^Significantly different between the 2 °C and 37 °C trials (*p* < 0.05)

Energy intake differed among the trials (6.7 ± 1.8 MJ vs. 7.9 ± 2.3 MJ vs. 8.5 ± 3.2 MJ for the 2 °C, 37 °C and 60 °C trials, respectively; *p* = 0.009) (Fig. 1). Post hoc tests revealed that energy intake in the 2 °C trial was 19% and 26% lower than the 37 °C (*p* = 0.039, 95% CI 15.051–585.749) and 60 °C (*p* = 0.025, 95% CI 51.498–793.684) trials, respectively. The time taken to feel completely full in the 2 °C, 37 °C, and 60 °C trials was 18.2 ± 6.5 min, 21.8 ± 9.4 min, and 24.1 ± 11.9 min, respectively. The time taken to feel completely full was 5.9 min shorter in the 2 °C trial than the 60 °C trial (*p* = 0.046, 95% CI 0.086–11.768).

### Subjective appetite perceptions

**Fig. 2:**
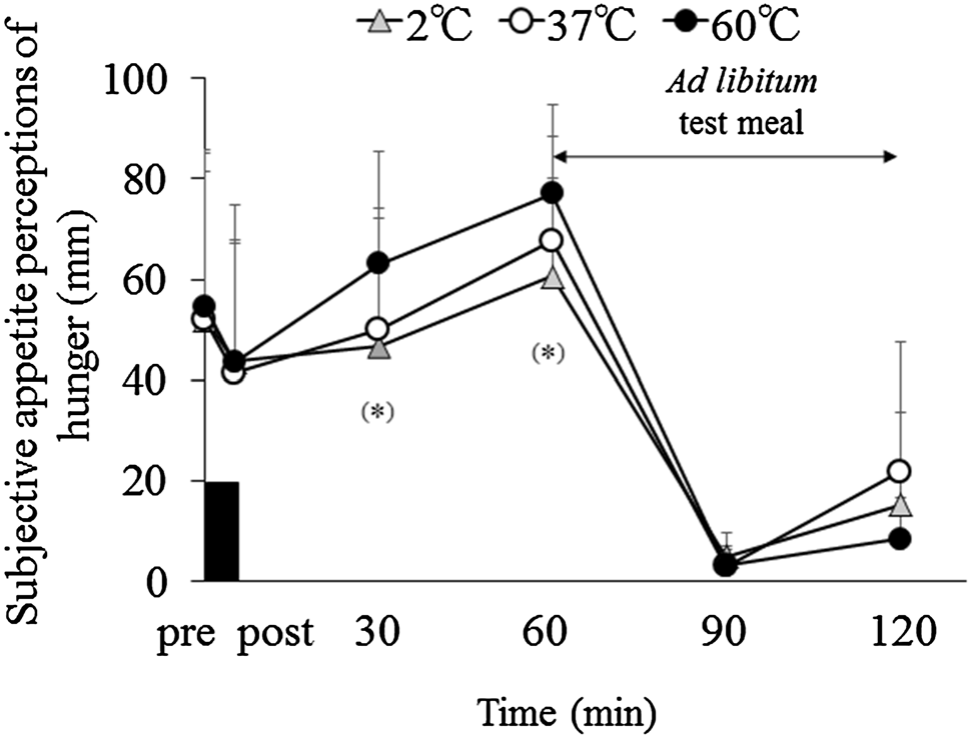
Subjective appetite perceptions of hunger before and after consuming water (500 mL) at 2 °C, 37 °C, and 60 °C. Data are mean ± SD. Black rectangle indicates consuming water in 5 min. Data were analyzed using two-factor ANOVA followed by a Bonferroni multiple-comparison test. There was a significant main effect of time (*p* < 0.001) and trial–time interaction (*p* = 0.027). (*)Different between the 2 °C and 60 °C trials (*p* < 0.10)

For subjective appetite perception of hunger, there was a significant main effect of time (*p* < 0.001) and trial–time interaction among the three trials (*p* = 0.027) (Fig. 2). Post hoc analysis revealed that subjective appetite perception of hunger tended to be lower during the 2 °C trial than the 60 °C trial at 30 and 60 min after consuming water (30 min: *p* = 0.074, 95% CI −1.557 to 38.557, 60 min: *p* = 0.086, and 95% CI −2.318 to 40.984). In each trial, subjective appetite perception of hunger peaked 60 min after consuming water (main effect of time: *p* < 0.001). There were no significant differences in the other subjective appetite perceptions (i.e., hunger and desire to eat sweet, sour, fatty, and salty foods) or perception of the stomach (i.e., “Dose your stomach feel uncomfortable?”, “Do you feel your stomach is expanding?”, and “Do you want to eat now?”).

### Gastric antral area and gastric contractions

**Fig. 3:**
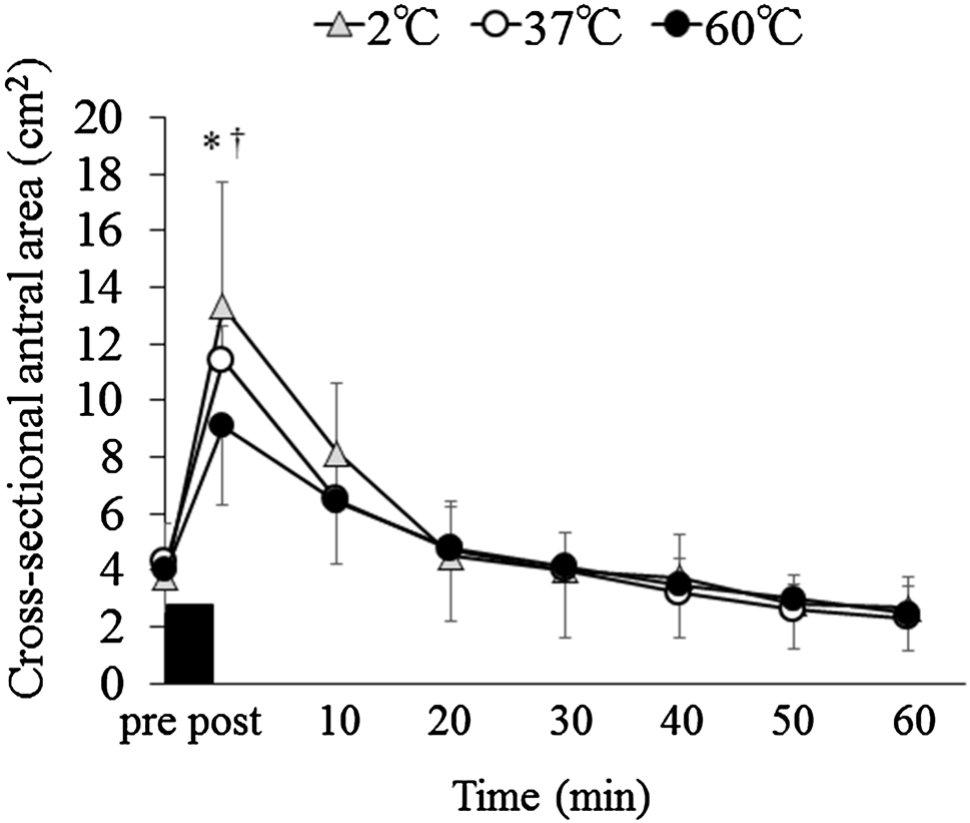
Cross-sectional gastric antral area before and after consuming water (500 mL) at 2 °C, 37 °C and 60 °C. Data are mean ± SD. Black rectangle indicates consuming water in 5 min. Data were analyzed using two-factor ANOVA followed by a Bonferroni multiple-comparison test. There was a significant main effect of time (*p* < 0.001) and trial–time interaction (*p* = 0.020). *Significantly different between the 2 °C and 60 °C trials (*p* < 0.05). ^†^Significantly different between the 37 °C and 60 °C trials (*p* < 0.05)

**Fig. 4:**
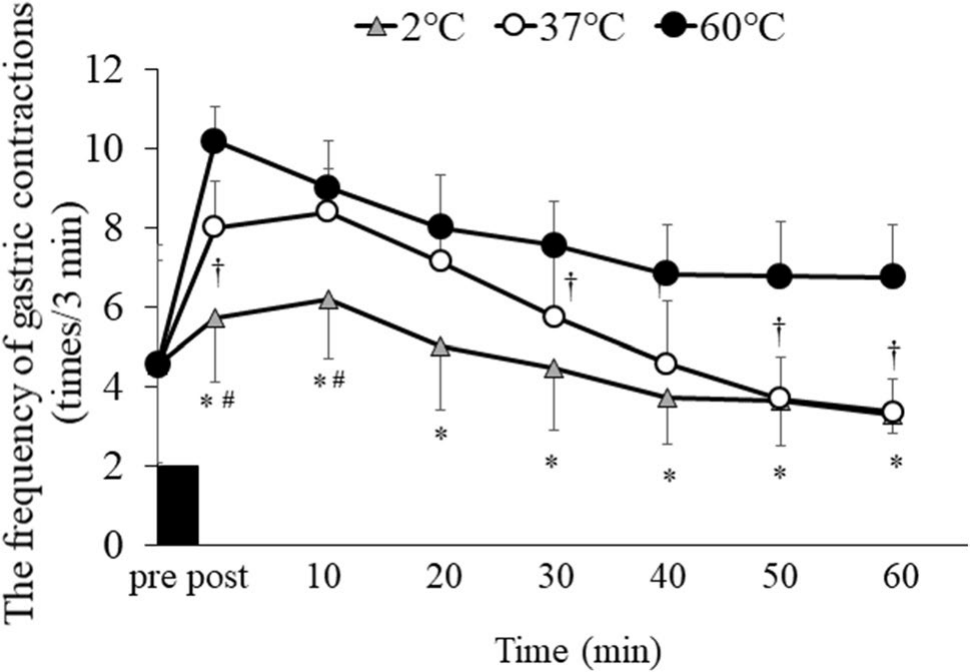
Frequency of gastric contractions before and after consuming water (500 mL) at 2 °C, 37 °C, and 60 °C. Data are mean ± SD. Black rectangle indicates consuming water in 5 min. Data were analyzed using two-factor ANOVA followed by a Bonferroni multiple-comparison test. There was a significant main effect of trial (*p* < 0.001), main effect of time (*p* < 0.001), and trial–time interaction (*p* = 0.020). *Significantly different between the 2 °C and 60 °C trials (*p* < 0.05). ^#^Significantly different between the 2 °C and 37 °C trials (*p* < 0.05). ^†^Significantly different between the 37 °C and 60 °C trials (*p* < 0.05)

There were trial–time interactions (*p* < 0.001). Cross-sectional antral areas increased in the 2 °C and 37 °C trials compared with the 60 °C trial immediately after consuming water (i.e., post) (10.0 ± 5.0 cm^2^ vs. 9.3 ± 1.2 cm^2^ vs. 6.8 ± 2.1 cm^2^ for the 2 °C, 37 °C and 60 °C trials, respectively; 2 °C vs. 60 °C: *p* = 0.030, 95% CI 0.379–7.802, 37 °C vs. 60 °C: *p* = 0.0019, 95% CI 0.407–4.556) (Fig. 3). The frequency of gastric contractions differed significantly among the trials (main effect of trial, *p* < 0.001, and trial–time interaction: *p* < 0.001). Post hoc analyses indicated differences in the frequency of the gastric contractions between trials immediately after (i.e., post) and at 10, 20, 30, 40, 50, and 60 min after consuming water—the frequency of the gastric contractions was lower in the 2 °C trial than the 60 °C trial (post: *p* < 0.001, 95% CI 2.818–5.932, 10 min: *p* = 0.001, 95% CI 1.202–3.548, 20 min: *p* = 0.004, 95% CI 1.158–4.592, 30 min: *p* = 0.002, 95% CI 1.333–4.167, 40 min: *p* = 0.016, 95% CI 0.512–4.238, 50 min: *p* = 0.026, 95% CI 0.341–4.909, 60 min: *p* = 0.002, 95% CI 1.608–5.142) (Fig. 4). At immediately after (i.e., post), and at 30, 50, and 60 min after consuming water, the frequency of the gastric contractions was lower in the 37 °C trial than the 60 °C trial (post: *p* = 0.011, 95% CI 0.605–3.895, 30 min: *p* = 0.034, 95% CI 0.104–2.371, 50 min: *p* = 0.024, 95% CI 0.369–4.656, 60 min: *p* = 0.005, 95% CI 1.192–5.408). At immediately after (i.e., post) and at 10 min after consuming water, the frequency of the gastric contractions was lower in the 2 °C trial than the 37 °C trial (post: *p* = 0.003, 95% CI 0.880–3.370, 10 min: *p* < 0.001, 95% CI 1.453–2.872).

### Relationships between energy intake and frequency of the gastric contractions

There was a positive relationship between energy intake from the test meal and the frequency of the gastric contractions measured immediately after consuming water. The reduction in frequency of the gastric contractions measured immediately after consuming water was associated with a reduction in energy intake (*r* = 0.365, *p* = 0.037).

## Discussion

The present study is, to our knowledge, the first to investigate how different water temperatures influence gastric motility and energy intake using ultrasound imaging systems. The main findings of the present study are that consuming 500 mL of water at 2 °C suppressed gastric contractions and ad libitum energy intake compared with consuming 500 mL of water at 37 °C and 60 °C. Furthermore, subjective appetite perception of hunger tended to be lower after consuming 500 mL of water at 2 °C than after consuming 500 mL of water at 60 °C. In addition, reduced energy intake after consuming cold water (i.e., at 2 °C) ingestion was accompanied by a change in gastric contractions. These findings add new knowledge to the existing literature that water temperature may play an important role in modulating gastric motility and energy intake. Although these findings need to be confirmed in larger and more diverse populations, the present study is of value in showing that pre-meal cold (vs. warm) water ingestion may provide additional benefits to weight management in healthy individuals as this method can be applied inexpensively and easily on a daily basis.

Three laboratory-based studies [5–7] have demonstrated reductions in energy intake after acute water ingestion which were seen in the present study. One study found that pre-meal (i.e., 30 min) water ingestion was effective in reducing subsequent energy intake in overweight and obese older adults [7]. Another study found similar results in response to pre-meal (i.e., 30 min) water ingestion in healthy older adults, but not in healthy young adults [5]. One recent study found that water ingestion immediately before a meal was effective in reducing subsequent energy intake in healthy young adults [6]. Although the precise reasons for the discrepant findings observed in the study by Van Walleghen et al. [5] are not known, the timing of ad libitum energy intake in response to water ingestion may affect subsequent energy intake [17, 18]. Indeed, a previous study has reported that inter-meal interval between drink ingestion and ad libitum meal was associated with gastric antral area and energy intake, although nutrient drink was used in this study [17, 18]. Another reason for the discrepant findings is that differences in the rate of gastric emptying between young and older individuals might affect subjective energy intake [6, 19]. Alternatively, given the well-documented slower rate of gastric emptying at cold water (i.e., at 5 °C) compared with warm water (i.e., at 37 °C) [20], the temperature of ingested water may be the proposed reason for the discrepant findings among the studies. Indeed, among three available studies examined the effect of pre-meal water ingestion on energy intake in humans [5–7], only one study has clearly mentioned the temperature of water used in the study (i.e., at 5–7 °C) [7].

The most plausible explanation for why different temperatures of water affected ad libitum energy intake is likely to be the changes in gastric motility. In the present study, the cross-sectional gastric antral areas measured immediately after consuming water (i.e., post) increased significantly in the 2 °C and 37 °C trials compared with the 60 °C trial. On the other hand, the frequency of the gastric contractions measured over 1 h after consuming water was lowered in the 2 °C trial than the 60 °C trial. The temperature of a drink is known to be one of the major factors affecting the gastric motility [8, 9, 21, 22]. Although no studies have addressed the effects of different water temperatures on the cross-sectional antral area, Mishima et al. [9] reported that the values of the lag-phase time, as an index of gastric emptying, were shorter after consuming nutrient drinks at 60 °C than consuming the same drinks at 37 °C. Several studies have suggested that gastric distention is one of the key factors in the regulation of energy intake [23–25]. The previous study has reported that subjective appetite perception of fullness was linearly related to the total gastric volumes [23]. Other studies have also reported that gastric distension prior to a meal is related with energy intake [10, 17]. In addition, there was a relationship between the frequency of the gastric contractions measured at immediately after consuming water and energy intake as demonstrated in the present study. One possible mechanism for these changes in gastric contractions associated with subjective energy intake is changes in gut hormones including motilin, a hormone that stimulates proximal stomach tone and enhances meal-induced satiety [26]. Future studies are required to address a more mechanical understanding of water temperature-induced modulation of gastric motility and energy intake. Furthermore, internal and external changes in temperature affect the physiological response in humans. Boschmann et al. have reported that consuming water induced thermogenesis with the stimulation of the sympathetic nervous system and the temperature of water in healthy adults [27]. In addition, the previous studies have addressed that exercising in a cold environment (2 °C) increased energy intake compared with a neutral condition (20 °C) in overweight adults [28], and fluid temperature affected gastric emptying rate in healthy adults [9]. Nonetheless, no studies are available to compare directly the effects of water-induced thermogenesis on gastric motility and appetite. Thus, further studies are warranted to examine these issues.

This study has several strengths. We examined the effects of different temperatures of water on both gastric motility and energy intake. The physical characteristics of the subjects, the amount of water drunk, and the time interval between ingestion of water and the subsequent meal were often used to address the factors for the influence of pre-meal water ingestion on energy intake [5–7]. To our knowledge, the present study is the first to examine the effects of different temperatures of water on subsequent energy intake. Moreover, we have tried to address the role of gastric motility, a potential mechanism underpinning the modulation of energy intake [23], on subsequent energy intake. The findings of the present study may help to support appetite adjustment in healthy young individuals as an inexpensive and easy method of weight management. The limitations of the present study include measuring gastric distention and contractions as the only indices of gastric motility. Future studies should examine how different water temperatures affect appetite-regulating hormones, including acylated ghrelin and peptide YY, and the effect of gastric blood flow on subsequent energy intake—appetite-regulating hormones and gastric blood flow are known to influence appetite and/or gastric motility [29, 30]. Furthermore, our findings are from an acute experimental study without a no-pre-meal water condition, indicating that our methodology has lack of a strong internal validity for assessing energy intake. Since a 12-week of pre-meal water ingestion has a positive impact on weight reduction in overweight/obese middle-aged and older adults [31], and obese middle-aged adults [32], it would be interesting for future studies to examine the effects of long-term pre-meal water ingestion at different temperatures on weight management in various populations. In addition, since the present study did not ask or objectively measure daily fluid intake for each subject, this habitual fluid intake and hydration status might play a role in subjective appetite [33] and energy intake. Thus, further studies should examine the effects of daily fluid intake and hydration status on gastric motility and energy intake.

In conclusion, this study demonstrates that gastric contractions and ad libitum energy intake were dependent on the temperatures of pre-meal water in healthy young men—consuming 500 mL of water at 2 °C 1 h before a meal was more effective in reducing gastric contractions and ad libitum energy intake than consuming the same amount of water at 37 °C and/or 60 °C. The present findings also show that cold water-induced reduction in energy intake appears to be related to the modulation of the gastric motility.
